# Fecal Transmission of Spodoptera frugiperda Multiple Nucleopolyhedrovirus (SfMNPV; *Baculoviridae*)

**DOI:** 10.3390/v17030298

**Published:** 2025-02-21

**Authors:** Eduardo Ávila-Hernández, Cindy S. Molina-Ruiz, Juan S. Gómez-Díaz, Trevor Williams

**Affiliations:** 1 Instituto de Ecología AC (INECOL), Xalapa 91073, Veracruz, Mexico; aiajle124avila@gmail.com (E.Á.-H.); cindymolinarz@gmail.com (C.S.M.-R.); juan.gomez@inecol.mx (J.S.G.-D.); 2 Instituto Tecnológico Superior de Libres, Libres 73780, Puebla, Mexico

**Keywords:** *Alphabaculovirus*, fall armyworm, Lepidoptera, bioassay, qPCR, viral occlusion body, maize

## Abstract

The production of viable nucleopolyhedrovirus in the feces of infected lepidopteran larvae represents a poorly understood route for virus transmission prior to host death. In the present study, we examined the presence of viable virus in the feces of fourth-instar *Spodoptera frugiperda* larvae infected with the Nicaraguan isolate of Spodoptera frugiperda multiple nucleopolyhedrovirus (SfMNPV-NIC). Feces production increased in samples taken at 2 to 6 days post-inoculation but was significantly lower in infected insects compared to controls. Second instars experienced 3.9 to 68.3% of polyhedrosis disease following consumption of feces collected at 2–5 days post-inoculation, which subsequently fell to 29.1% in the 6-day sample. Calibration of the insect bioassay using OB-spiked samples of feces indicated that the concentration of OBs varied between 5.4 × 10^2^ and 4.4 × 10^5^ OBs/100 mg of feces in infected fourth instars. Quantitative PCR analysis of fecal samples indicated the presence of 0 to 7629 copies/mg feces following amplification targeted at the polyhedrin gene. However, no correlation was detected between qPCR estimates of virus concentration and time of sample collection or the quantity of feces collected. The qPCR estimates were positively correlated with the prevalence of lethal infection observed in the insect bioassay, but the correlation was weak and several samples did not amplify. Calibration of the qPCR assay using OB-spiked samples of feces provided estimates that were 5- to 10-fold lower than the insect bioassay, indicating inhibition of the amplification reaction or loss of material during processing. In a greenhouse experiment, 2.5–48.3% of second-instar larvae acquired lethal infection following a 24 h period of feeding on maize plants on which fourth instar larvae had deposited their feces at 3 days and 4 days post-infection, respectively. These findings highlight the potential of OB-contaminated feces as a source of biologically significant quantities of inoculum for virus transmission prior to the death of infected insects and represent an additional contribution to the biological control of lepidopteran pests by these pathogens.

## 1. Introduction

In most cases, lepidopteran nucleopolyhedroviruses (*Alphabaculovirus*, *Baculoviridae*) transmit horizontally through viral occlusion bodies (OBs) released from the cadavers of diseased larvae [1,2]. The OBs contaminate foliage and can persist in the environment on plant surfaces or in soil for extended periods [3].

Host larvae that consume contaminated foliage acquire an infection when OBs dissolve in the alkaline midgut and release occlusion-derived virions (ODVs) that cross the peritrophic matrix and infect midgut cells [4], a process that is mediated by a specific complex of per os infection factors present in the ODV envelope [5]. Once inside the cell, viral nucleocapsids are transported to the nucleus by the actin cytoskeleton to initiate replication, whereas others may be immediately repackaged and exported as budded virions (BV) across the basal membrane to infect the cells of the tracheal system, hemocytes, and other tissues, the mechanisms of which have been reviewed in great detail elsewhere [6]. These stages of infection are known as the primary and secondary (systemic) stages, respectively.

Most insect midgut studies have focused on the progression of the infection and the establishment of the systemic infection [7,8], or the role of the midgut epithelium as the first determinant of host range [9,10,11]. On occasions, it has also been noted that midgut cells can produce OBs following primary infection [12,13,14]. These productively infected cells may be sloughed off and expelled into the midgut lumen as a means of limiting the progression of the infection [15,16,17]. Modern transcriptomic studies have provided strong additional evidence that the midgut cells are a highly active site of nucleopolyhedrovirus replication [18,19].

Given the involvement of midgut cells in the primary infection process, it is not unsurprising that a small number of studies have detected the presence of viable virus in larval feces at various time points post-infection [20,21,22] or viral DNA in frass collected from beneath host plants [23]. In *Mamestra brassicae* and *Anticarsia gemmatalis*, infected larvae that traveled over host plants have also been demonstrated to contaminate leaf surfaces with their homologous nucleopolyhedroviruses prior to death. Indeed, leaf surfaces contaminated by larval feces can harbor sufficient virus to initiate infections in healthy conspecifics that subsequently inhabit the same plants [21,24].

In view of the rapid expansion in the geographical range of the fall armyworm, *Spodoptera frugiperda* across tropical and subtropical regions of Africa, Asia, and Oceania in recent years [25], the need to identify effective and sustainable control strategies has become increasingly evident [26]. The Spodoptera frugiperda multiple nucleopolyhedrovirus (SfMNPV) has attracted considerable attention as the basis for biological insecticides targeted at this pest in its native range [27,28] and its expanded range [29,30]. A Nicaraguan isolate of this virus has been extensively studied for its insecticidal properties and genotypic composition. This isolate comprises a mixture of at least nine genotypic variants that vary their prevalence and insecticidal phenotype [31]. Only one of the variants comprises the complete genome (SfNIC-B), whereas the remaining variants have deletions of up to 16.3 kb, mainly located in a region rich in auxiliary genes [32]. The Nicaraguan isolate is closely related to an isolate of this virus from Argentina, but shows greater divergence from SfMNPV isolates from Colombia, Brazil, and the USA [33].

In the present study, we hypothesized that fecal contamination of plant surfaces could be a previously unrecognized source of transmission in the fall armyworm—SfMNPV pathosystem. To address this, we examined the production of OBs in the feces of larvae at different intervals post-inoculation. We also estimated the concentration of viable OBs in the feces using bioassay and quantitative PCR techniques and evaluated feces-mediated transmission of the pathogen in a greenhouse experiment.

## 2. Materials and Methods

### 2.1. Insect Colony and Virus

Larvae of *S. frugiperda* were obtained from a laboratory colony originating from maize fields in Veracruz State, Mexico (19.43745° N, −96.37787° W). The larvae were reared at 26 ± 1 °C, 70 ± 10% relative humidity, a 14:8 h L:D photoperiod and fed on a semi-synthetic diet mainly containing wheatgerm, soybean, maize flour, and yeast [34]. The colony was known to be free from sublethal SfMNPV infection [35].

The Nicaraguan wild-type isolate (SfMNPV-NIC) was grown by inoculating fourth instars with 1 × 10^8^ OBs/mL (equivalent to a 99% lethal concentration) using the droplet-feeding method. Larvae were starved overnight prior to inoculation. Inoculated larvae were reared individually on semi-synthetic diet at 27 ± 0.5 °C in darkness until death. Virus-killed insects were macerated in ultrapure water (GenPure xCAD Plus, Thermo Fisher Scientific, Waltham, MA, USA) and filtered through an 80 μm steel mesh. The resulting suspension was centrifuged at 400× *g* for 10 min to sediment debris, passed through a 40% glycerol cushion at 5900× *g* for 15 min, washed once with ultrapure water, and resuspended in 500 μL of ultrapure water. Triplicate samples of the OB suspension were then counted in an improved Neubauer chamber (×400) and stored at 4 °C until required for experiments.

### 2.2. Production of Virus-Contaminated Feces

Fourth instars of *S. frugiperda* were starved overnight and then inoculated with 1 × 10^8^ OB/mL of SfMNPV-NIC in 10% sucrose solution using the droplet-feeding technique. This inoculum concentration was expected to result in >95% lethal polyhedrosis disease. Control larvae were inoculated with sucrose solution alone. Larvae that drank the OB suspension within 15 min were individually transferred to the wells of a 12 well tissue culture plate with a piece of diet and were incubated in darkness at 27 ± 0.5 °C. After 24 h, the larvae were believed to have voided any residual OB inoculum and were rinsed with 0.1 M sodium carbonate solution and distilled water and individually transferred to a clean 12-well plate. Wells contained a layer of diet, 5 mm deep, covering the bottom of each well. Nine inoculated larvae and three control larvae were placed in each plate, which comprised one replicate. The plate was sealed with a paper towel and a ventilated lid. Each plate was placed on its longest edge so that larvae would feed vertically and feces would accumulate on the side-wall of each well. Preliminary tests indicated that the vertical orientation of plates facilitated the easy recovery of feces from each well using a toothpick. Plates were incubated in darkness at 27 ± 0.5 °C and at 24 h intervals the feces were collected from each well, placed in a preweighed microcentrifuge tube, weighed to an accuracy of ±1 mg, and were frozen at −20 °C until required. If a larva was found to have died over the previous 24 h period, a fecal sample was not taken to avoid potential contamination issues arising from OBs released from virus-killed insects. The experiment was replicated six times using different batches of larvae.

To estimate the water content of feces produced by diet-fed larvae, feces samples were collected from fourth instars (n = 69) after a 24 h feeding period, placed in pre-weighed microcentrifuge tubes and individually weighed on a precision balance (Explorer EX124, Ohaus Corp., Parsippany, NJ, USA) to determine wet weight. The tubes were then opened and placed in an oven at 60 °C for 24 h, then reweighed to determine the dry weight.

### 2.3. Presence of Virus in Fecal Samples: Insect Bioassay

The detect the presence of viable OBs in feces, each sample was pooled for all the infected or control larvae from a particular sample date within each replicate and diluted in a volume of 10% sucrose that was 10-fold the fresh weight of the feces, e.g., a pooled sample of 150 mg of feces would be diluted in 1.5 mL of sucrose solution. This dilution factor was necessary because of the paste-like consistency of the feces. Second, instars of *S. frugiperda* were starved overnight and then allowed to drink feces samples in sucrose solution. A group of 24 larvae was inoculated with each feces sample and was then incubated individually in the wells of a 24-well cell culture plate with a piece of diet at 27 ± 0.5 °C. The larvae were checked daily for deaths due to polyhedrosis disease. In case of doubt, larval tissue was smeared on a microscope slide, treated with Giemsa stain, and examined for the presence of OBs under a phase-contrast microscope. Each fecal sample was bioassayed on three groups of 24 larvae from different batches of insects representing three replicates.

To calibrate the sensitivity of the bioassay system, fecal samples (100 mg feces in 1 mL of 10% sucrose solution) from healthy fourth instars were spiked with one of six concentrations of OBs ranging from 3.3 × 10^7^ to 3.3 × 10^2^ OBs/mL produced by serial dilution of the stock OB suspension or a blank control. The spiked samples were subjected to bioassay on groups of 24 *S. frugiperda* second instars following the procedures described for the fecal samples from infected insects. The spiked-sample bioassay was replicated on four occasions using different batches of larvae.

### 2.4. Presence of Virus in Fecal Samples: Quantitative PCR

To estimate the concentration of virus genomes present in feces, quantitative PCR (qPCR) was performed on DNA extracted from fecal samples. As feces had previously been diluted in a 10% sucrose solution for the bioassay (Section 2.3), a 1 mL volume of each sample (containing 100 mg of feces) was taken from 12 randomly selected fecal samples collected at 3–6 days post-inoculation and 3 randomly selected control samples (collected at 3–4 days). Each suspension was centrifuged at 3300× *g* for 15 min to sediment feces and OBs. The supernatant was removed and discarded, and 400 µL of ultrapure water and 200 µL of 3 × DAS buffer (0.51 M NaCl, 0.3 M Na_2_CO_3_, 0.03 M EDTA) were added to resuspend the pellet. The suspension was incubated at 38 °C for 30 min to dissolve OBs and release ODVs, followed by centrifugation at 1500× *g* for 12 min to pellet feces and undissolved OBs. The supernatant was recovered, diluted in an equal volume of water, and centrifuged at 16,000× *g* for 20 min to pellet ODVs. The supernatant was then removed, the pellet was resuspended in 200 µL of water, and DNA was recovered using the DNAeasy Blood and Tissue Kit (Qiagen, Hilden, Germany). The resulting DNA was suspended in 40 μL of ultrapure water and used for PCR analysis.

qPCR was performed following the optimized protocol described by Molina-Ruiz et al. [34]. Briefly, reaction mixtures were prepared as follows: 5 μL of iQ SybrGreen 2× MasterMix (BioRad, Hercules, CA, USA), 0.5 μM of each primer, 2.4 μL ultrapure water, and 2 μL of the sample DNA (comprising 2.6 to 15 ng of total DNA depending of the sample). The primers targeted the *polyhedrin* gene (polhFw 5′-GCCCGTGTACGTAGGAAACA-3′; polhRv 5′-ACTCTTCGAAGGAGTGCGTG-3′) to produce a predicted amplicon of 110 bp. Reactions were prepared in triplicate. Water was used as the negative control. The reaction cycle consisted of 3 min at 95 °C, followed by 40 cycles of 30 s at 95 °C, 30 s at 60 °C, and 1 min at 72 °C using a Stratagene Mx3005p qPCR thermocycler (Agilent, Santa Clara, CA, USA). The lower limit of detection of this technique was previously determined at 10 copies/reaction [34], so that samples that produced lower copy number estimates were taken to be zero.

To calibrate the sensitivity of the qPCR assay, samples of 100 mg feces from healthy fourth and fifth instars of *S. frugiperda* were spiked with serial dilutions ranging from 5 × 10^4^ to 5 × 10^7^ OBs of SfMNPV-NIC in 1 mL of 10% sucrose solution (as previously used for analysis of the bioassay samples). Four replicate samples of each dilution were prepared, subjected to DNA extraction and finally recovered in a volume of 40 µL using the DNAeasy Blood and Tissue Kit spin columns (Qiagen, Hilden, Germany). A 2 µL volume of each sample was used in each reaction, plated in triplicate, with blank controls and subjected to qPCR amplification.

### 2.5. Transmission Under Greenhouse Conditions

**Figure 1:**
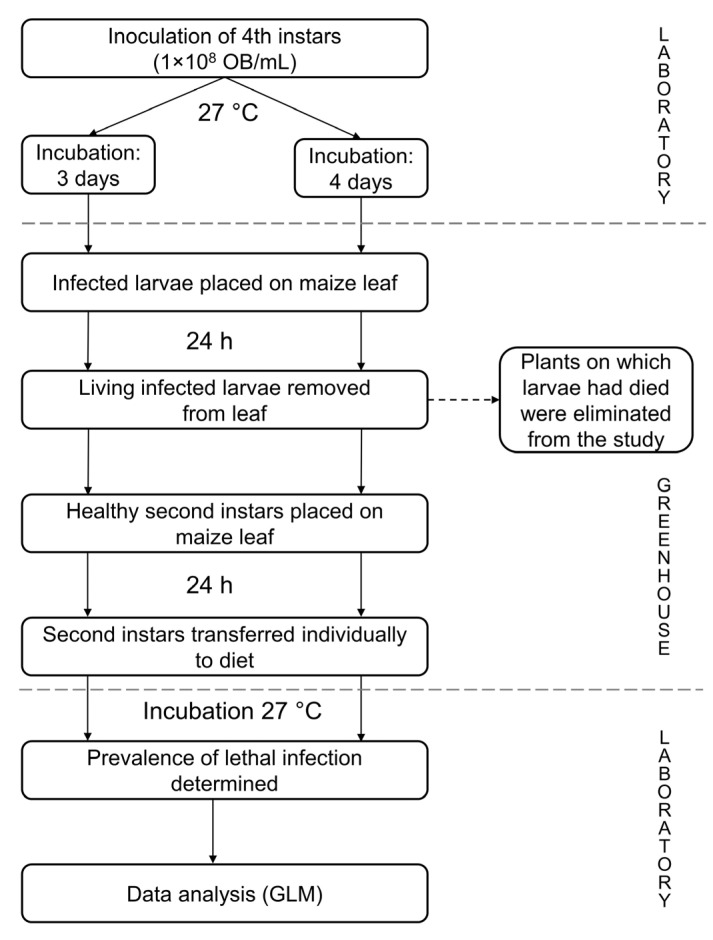
Schematic of the steps performed to evaluate transmission of SfMNPV in feces of infected fourth instars to healthy second instars on maize plants under greenhouse conditions.

Maize plants (*Zea mays*) hybrid variety H-318 (Codegram, Tenencia, Mexico) were grown individually in soil in 250 mL polystyrene cups in a greenhouse. A generic granular fertilizer (NPK 17:17:17) was applied when plants were approximately 10 cm in height. Fourth instars of *S. frugiperda* were inoculated with 1 × 10^8^ OBs/mL and incubated for either three or four days at 27 ± 0.5 °C, as described in Section 2.2. Control larvae were treated identically but were inoculated with sucrose solution alone. At three or four days post-inoculation (Figure 1), larvae were placed individually on the leaf of a maize plant (30–40 cm height) and confined within a cage comprising a plastic Petri dish cage with a 3 mm layer of polyethylene–vinyl acetate sponge as the floor. The dish was held together with an elastic band so that each larva was exposed to a 9 cm length of leaf for a period of 24 h. Plants on which larvae died from polyhedrosis disease during the 24 h greenhouse period were excluded from the experiment. For larvae that were alive after this period, each infected larva was removed, and a group of four second-instar larvae was placed on the exposed leaf section for an additional period of 24 h (Figure 1). To determine the prevalence of virus transmission, larvae were then transferred individually to the wells of a 24-well cell culture plate with a piece of diet, taken to the laboratory and incubated at 27 ± 0.5 °C until death or pupation. Virus-induced deaths were confirmed by observation of OBs in Giemsa-stained smears of larval tissues. The prevalence of lethal polyhedrosis disease in the second instars that were exposed to the feces of infected fourth-instar larvae was subjected to statistical analysis (Section 2.6).

Of the 3-day and 4-day post-infection larvae, a total of 35 and 65 larvae were inoculated, respectively, but differential mortality while feeding on maize meant that 5 and 37 larvae, respectively, were eliminated from the experiment, leaving a total of 30 and 29 larvae (replicates), respectively. A total of 16 mock-infected control larvae were included in the study to control for accidental contamination.

### 2.6. Statistical Analyses

All data were checked for normality and homoscedasticity prior to analysis by using the Shapiro–Wilk test and Levene’s test, respectively. The weight of feces per larva was averaged within each replicate, normalized by √(*x* + 0.5) transformation and subjected to repeated measures analysis of variance (ANOVA) for samples collected at 2–6 days post-inoculation. The mortality values of *S. frugiperda* second instars that consumed feces from infected fourth instars were normalized by √(*x* + 0.5) transformation prior to one-way ANOVA. Data on qPCR quantification of virus genomes were normalized by log (*x* + 1) transformation prior to regression analysis. The prevalence of lethal infection of second instars exposed to feces in the greenhouse experiment was compared by fitting a generalized linear model (GLM) with a binomial error distribution and logit link function. The standard errors (SE) of binomially distributed means become increasingly asymmetrical as they approach the limits of the distribution and are therefore presented as the upper and lower range. All analyses were performed using the R-based software Jamovi v.2.5 [36].

## 3. Results

### 3.1. Production of Virus-Contaminated Feces

**Figure 2:**
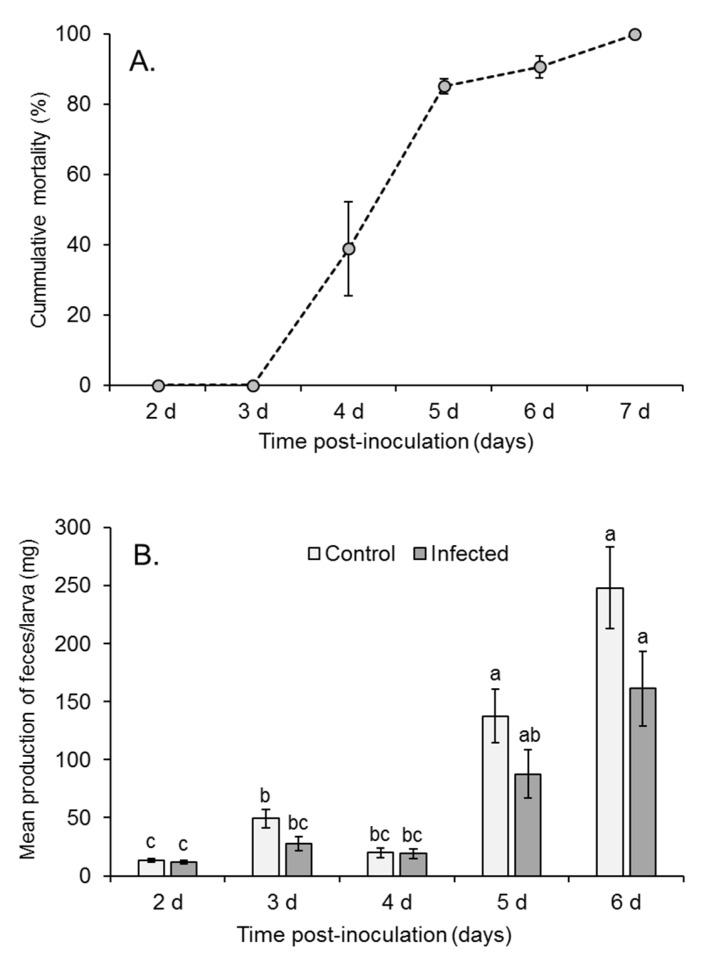
(**A**) Mean (±SE) cumulative mortality of fourth instars of *Spodoptera frugiperda* at 2–7 days post-inoculation. (**B**) Mean (±SE) fresh weight of feces collected from control and infected larvae at 2–6 days post-inoculation. Samples were not collected on the day of death of larvae to avoid contamination from OBs released from insect cadavers. Columns headed by identical letters did not differ significantly (repeated measures ANOVA, Tukey *p* > 0.05).

Virus-induced death of inoculated fourth instars was first observed at 4 days post-inoculation and increased until all larvae had died by 7 days post-inoculation (Figure 2A). None of the control larvae died from polyhedrosis disease.

The daily production of feces varied significantly during the experiment (F = 25.881; df = 4, 40; *p* < 0.001). Feces production increased slightly between 2 and 3 days post-inoculation but decreased on day 4 as larvae molted to the fifth instar, although the majority of these changes were not statistically significant (Figure 2B). The production of feces then increased markedly at 5 and 6 days post-inoculation, as fifth instars are voracious feeders. No samples were taken at 7 days post-inoculation, as feces were not sampled on the day of the death of the larvae to avoid possible contamination from OBs released from virus-killed insects. Overall, infected larvae produced significantly lower quantities of feces compared to control insects, presumably due to reduced feeding in diseased insects (time*treatment interaction F = 3.664; df = 4, 40; *p* = 0.012). The fecal samples had a mean (±SE) water content of 45.6 ± 2.9% (n = 69 samples).

### 3.2. Presence of Virus in Fecal Samples: Insect Bioassay

**Figure 3:**
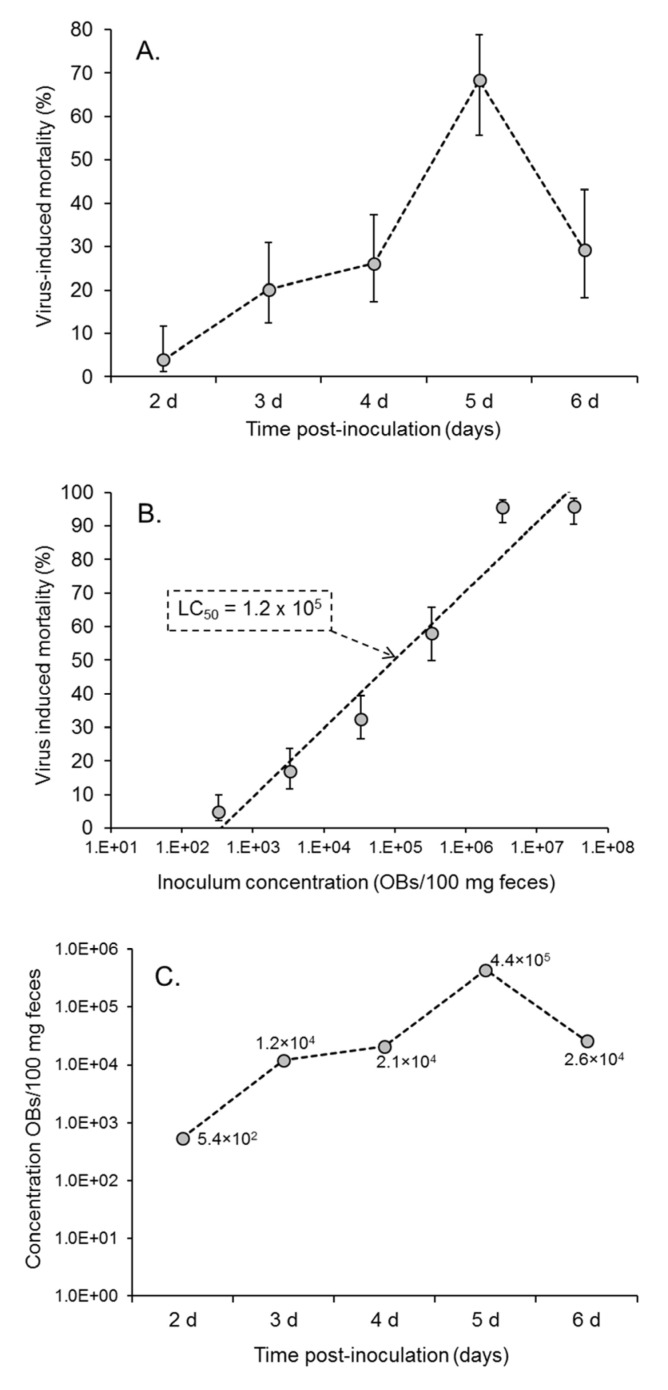
Results of bioassay of fecal samples in *S. frugiperda* larvae: (**A**) Mean (±SE) virus-induced mortality of second instars that consumed fecal samples collected from infected larvae at 2–6 days post-infection. (**B**) Mean (±SE) virus induced mortality of second instars inoculated with 100 mg feces spiked with serial dilutions of OBs. The 50% lethal concentration (LC_50_) is indicated on the graph. (**C**) Estimated concentration of OBs present in fecal samples shown in (**A**) based on the concentration-mortality results shown in (**B**).

The mean (±SE) mortality of second instars that consumed diluted suspensions of feces varied significantly with the time of feces collection (F = 5.524; df = 4, 22; *p* = 0.003) (Figure 3A). Virus-induced mortality increased from 3.9% (range of SE: 1.2–11.8%) in samples taken at 2 days post-inoculation to a maximum of 68.3% (range of SE: 55.6–78.8%) in samples taken at 5 days post-inoculation. Mortality then decreased in the final sample, although the final sample originated from a low number of larvae that were still alive in the day 6 sample (shown in Figure 2A). None of the insects that consumed feces from control larvae died from polyhedrosis disease.

The bioassay of 100 mg fecal samples from healthy larvae that were spiked with different quantities of OBs resulted in a positive correlation between inoculum concentration and prevalence of virus-induced mortality (F = 129.8; df = 1, 20; *p* < 0.001) (Figure 3B). The logit regression equation was *y* = 1.357(±0.1686)*x* − 6.894, where *y* is Ln(*p*/*q*), in which *p* is the proportion of virus-induced mortality, and *q* is the proportion of larvae that did not succumb to disease. Using this equation, the concentration of OBs in the fecal samples from infected larvae was estimated and increased from 5.4 × 10^2^ OBs/100 mg feces (in 1 mL of fecal suspension) in the 2-day samples to a maximum of 4.4 × 10^5^ OBs/100 mg feces in the 5-day samples (Figure 3C).

### 3.3. Presence of Virus in Fecal Samples: Quantitative PCR

**Figure 4:**
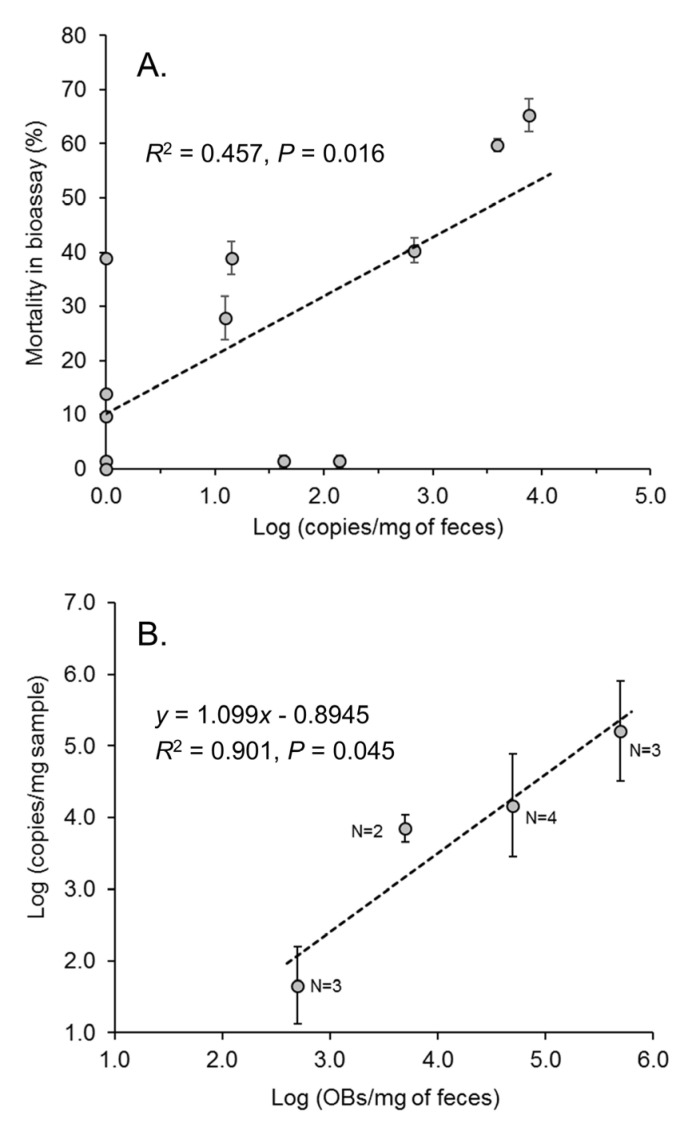
qPCR quantification of SfMNPV genome copies in fecal samples: (**A**) Correlation of logarithm of number of genome copies/mg of feces and the mean (±SE) percentage of virus-induced mortality of *S. frugiperda* second instars that were inoculated with fecal samples. (**B**) Estimates of genome copies/mg of fecal samples spiked with 5 × 10^2^–5 × 10^5^ OBs/mg. Numbers next to data points indicate the number of replicate samples that amplified in the qPCR assay.

Control feces were all negative for viral DNA with Ct values of >33. Estimates of the concentration of viral DNA in fecal samples from virus-inoculated larvae ranged from 0 to 7629 copies/mg feces. The quantity of virus genomes/mg feces did not increase significantly in samples taken at 3 days post-inoculation and those taken at later time points (R^2^ = 0.001, *p* = 0.909) (Supplemental Figure S1A). There was no significant correlation between fecal sample weight and the concentration of viral genomes/mg, indicating that the viral DNA was not more diluted in samples of greater weight (R^2^ = 0.14, *p* = 0.231) (Supplemental Figure S1B). There was, however, a significant correlation between the concentration of viral genomes/mg of feces and the mortality recorded in *S. frugiperda* second instars that were inoculated with fecal samples in the insect bioassay (Figure 4A). Nonetheless, a considerable variation was present that included mortality (10–39%) in larvae that consumed samples containing near zero levels of viral DNA (below the qPCR quantification threshold) and samples containing tens or hundreds of viral genomes/mg that resulted in little or no mortality in inoculated insects.

To calibrate the qPCR assay in the presence of fecal material, samples of 100 mg of feces from healthy insects were spiked with 5 × 10^4^–5 × 10^7^ OBs (equivalent to 5 × 10^2^–5 × 10^5^ OBs/mg fecal sample). Despite attempting to remove contaminants from the resulting DNA samples, the DNA solution had a slight yellow tinge derived from the components of the larval diet. A pale tinge remained after samples were cleaned using silica spin columns. Of the four replicate samples of each OB concentration, between two and four replicate samples at each concentration were amplified; the remaining samples failed to amplify and were eliminated. A significant relationship was detected between the OB concentration in feces and the number of genome copies estimated in the qPCR assay (R^2^ = 0.901, *p* = 0.045) (Figure 4B).

Given that the feces samples from infected fourth instars that amplified by qPCR produced estimates of between 10^1.093^ and 10^3.883^ genome copies/mg feces (Figure 4A), using the calibration regression equation of Figure 4B, this range of values would be equivalent to 153–5250 OBs/mg feces, or 1.5 × 10^2^–5.3 × 10^4^ OBs/100 mg of feces. This range of estimates is approximately 5- to 10-fold lower than the estimate provided by the insect bioassay (Figure 3C) and suggests that fecal material resulted in an inhibition of the qPCR reaction and/or viral DNA was lost during the sample processing.

### 3.4. Transmission Under Greenhouse Conditions

**Figure 5:**
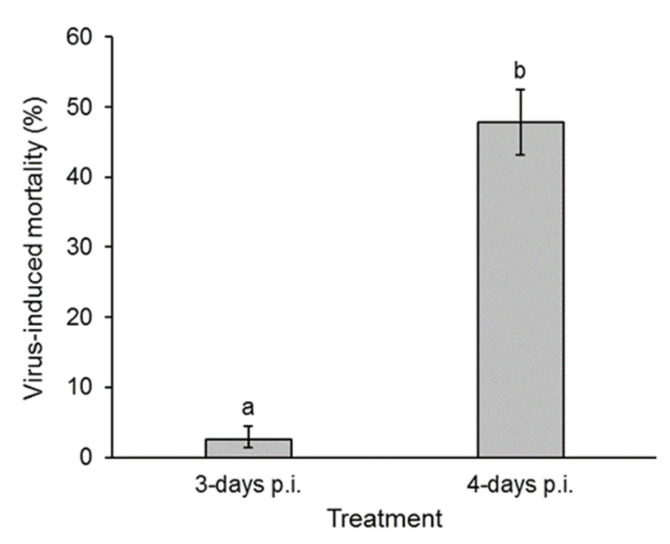
Mean (±SE) prevalence of lethal infection in *S. frugiperda* second instars that were exposed to the feces of fourth-instar larvae at 3 and 4 days post-inoculation (p.i.) on maize plants in a greenhouse experiment. Columns headed by different letters differ significantly (GLM, *p* < 0.05). No virus-induced mortality was observed in control insects.

Of the 3-day post-inoculation larvae used in the greenhouse experiment, 14% (5 larvae) died while feeding on maize plants, whereas 56% (36 larvae) of 4-day post-infection larvae died on maize plants. These insects were excluded from the experiment as they may have leaked OB-contaminated fluids following death. This resulted in a total of 30 and 29 replicates of infected but living larvae at 3 and 4 days post-inoculation, respectively. The prevalence of virus infection in second instars exposed to larval feces on maize plants varied significantly between the 3-day and 4-day post-infection treatments (GLM χ^2^ = 75.37, df = 1, *p* < 0.001). Of the 3-day post-inoculation samples, virus transmission was observed in only three replicates, and, in each case, only one out of the four second-instar larvae died from polyhedrosis disease (Figure 4). In contrast, virus transmission was observed in 27 out of 29 replicates, in which second instars were exposed to fecally contaminated maize plants that larvae had fed on at 4 days post-infection. Overall, 48% (range of SE: 43–52%) of these second instars subsequently died from polyhedrosis disease (Figure 5). Of the second instars that were exposed to feces of the control larvae on plants, none died of polyhedrosis disease.

## 4. Discussion

The sustained productive infection of midgut cells by nucleopolyhedroviruses is only seen in a fraction of the insect–nucleopolyhedrovirus pathosystems [37,38,39], so that the presence of biologically significant quantities of virus in lepidopteran feces that can be a source of subsequent transmission is not an inevitable consequence of each and every nucleopolyhedrovirus infection. In the present study, feces were collected from *S. frugiperda* larvae that had been inoculated with a lethal concentration of SfMNPV-NIC OBs in the fourth instar. The production of feces in these larvae increased markedly during the study as the larvae developed to the fifth instar. Feces production was significantly lower in infected larvae compared to the control, as observed previously in nucleopolyhedrovirus-infected *Chrysodeixis* (*Pseudoplusia*) *includens* on soybean [40], which reflects the reduced food consumption of diseased larvae, especially in the late stages of infection [41,42,43].

The bioassay of fecal samples in *S. frugiperda* second instars revealed the presence of a viable virus. The low prevalence of infection in larvae that consumed the 2 d post-inoculation samples confirmed a minimal carry-over of the original inoculum from the fourth-instar larvae. Similar levels of mortality (1.7–3.3%) were detected in *M. brassicae* larvae that consumed fecally contaminated semi-synthetic diet at 24 and 48 h post-inoculation [21].

The prevalence of mortality in the bioassay increased up to a maximum in the 5 d post-inoculation samples. Previous studies reported increases in virus in feces in the period 1–2.5 days post-inoculation in *Hyblaea puera* [22] and 1–4 days post-inoculation in *H. zea* [20]. Vasconcelos [21] also observed an increase 2–5 d post-inoculation followed by a decrease in virus-induced mortality of *M. brassicae* in 6 d and 7 d samples, as observed in the present study as few insects remain alive at the end of the study.

By calibrating the bioassay using spiked samples of feces, it was possible to estimate that the quantity of viable OBs in *S. frugiperda* feces increased by three logarithms between 2 and 5 days post-inoculation. Feces were not collected on the day of the death of the infected insect to avoid possible contamination from OBs originating from the liquefying body of the larva. Nonetheless, it seems likely that feces produced in the hours prior to death would also contain biologically significant OB loads. Although the quantities of OBs present in feces were approximately three logarithms lower than the quantities released following the death of an infected late instar larva (~10^8^ OBs), they are released over a period of several days and likely distributed over a wide area of the plant, both of which would contribute to preemptive transmission of the pathogen.

From a methodological perspective, it is important to note that the presence of fecal material interfered with laboratory assays in two ways. First, OBs mixed with feces resulted in an LC_50_ value of 1.2 × 10^5^ OBs/100 mg feces in 1 mL of suspension, which is approximately five-fold higher than previously estimated for the SfMNPV-NIC OBs in droplet-inoculated *S. frugiperda* second instars in our laboratory [44]. We assume this was due to the adsorption of OBs onto the surface of fecal material so that OBs were not uniformly distributed in the inoculum consumed by larvae during the bioassay. The diet-fed larvae also produced paste-like feces that had to be diluted 10-fold before they could be used in droplet-feeding bioassays. This would have reduced the sensitivity of the bioassay, especially for samples containing small quantities of virus.

Second, the qPCR quantification of genome copies in fecal samples was sub-optimal as samples did not amplify in several of the replicates. Despite spin-column treatment of DNA, the samples retained a pale yellow coloration carried over from the insect feces. qPCR-based estimates of the presence of OBs in fecal samples (1.5 × 10^2^–5.3 × 10^4^ OBs/100 mg of feces) were approximately 5- to 10-fold lower than the estimates provided by the insect bioassay. More importantly, there was no correlation between qPCR estimates and the prevalence of virus-induced mortality observed in insects that consumed feces from infected fourth instars. We attribute this to the presence of contaminants that inhibited the amplification and loss of virus material during sample processing. In a previous study, the sensitivity of qPCR for environmental samples with high contamination was improved by pre-amplification of the polyhedrin gene using end-point PCR and was used to detect the presence of nucleopolyhedrovirus in samples of feces collected from webworm nests (*Hyphantria* sp.), but the technique only yielded a positive or negative result rather than a quantitative estimate of viral genomes [23].

The greenhouse experiment on maize plants revealed that the levels of virus in feces were sufficient to contaminate plant foliage, resulting in 2.5% and 48% of virus-induced mortality in second instars of *S. frugiperda* exposed to foliage contaminated by 3-day and 4-day post-inoculation larvae, respectively (Figure 5). This compares to 13% mortality in *M. brassicae* second instars [21] on cabbage and 22% mortality in *A. gemmatalis* third instars on soybean [24] that acquired their respective homologous viruses from conspecific larval feces at 6 d post-inoculation.

The importance of the midgut in the production of OB-contaminated feces varies in each host–virus pathosystem. Early electron microscope studies revealed that OBs were never or rarely observed in midgut columnar cells [12,45,46] or only transiently [39,47], whereas OBs were plentiful in midgut cells of *A. gemmatalis*-AgMNPV at 120 h p.i. [14]. However, such direct observations may be hindered by the disappearance of infected midgut cells through the sloughing response that can occur as soon as 16 h p.i. in *Heliothis virescens* [16]. Larvae also empty the midgut lumen and expel sloughed infected cells immediately prior to each molt [16].

In a significant advance, single-nucleus RNA-sequencing techniques have now demonstrated that all cell types (classified based on shared gene expression patterns) present in the *Bombyx mori* midgut are infected with BmNPV at 72 h post-inoculation. However, the viral load varies markedly across different cell types with certain columnar cell types having obviously higher loads than goblet cells and intestinal stem cells, compared to intermediate loads in the endocrine cells and muscle cells [48]. The columnar cells are characterized by their abundant microvilli and are involved in absorption of nutrients and the synthesis and secretion of digestive enzymes [49]. The goblet cells in contrast are involved in mucus production as an epithelial barrier to pathogens and physical abrasion and are also primarily responsible for the alkaline pH via an active H^+^/K^+^ exchange mechanism, in combination with a carbonic anhydrase that generates a bicarbonate ion flux resulting in high pH [50,51].

We expect that all, or nearly all, of the viruses detected in fecal samples would be in the form of occluded ODVs rather than BVs. This is because the bioassay revealed the presence of orally infectious virus in feces. The BVs are several thousand folds less infectious per os than ODVs [52], as BVs lack the complex of PIF proteins required for midgut cell infection [53]. Furthermore, nucleocapsids produced in midgut cells are invariably trafficked by microtubules and actin polymerization to the basal membrane for release and are never observed budding through the microvilli of the lumen-facing cell membrane [38,54]. Moreover, of the viral genomes produced in each cell, most are occluded within OBs, whereas only a small fraction (~2%) are released as BVs [55], further supporting the idea that BVs probably comprise little if any of the viruses that we detected in feces.

The midgut is a purposefully detrimental environment for entomopathogens. Given this, the viral OBs or ODVs released from sloughed or degraded midgut cells will face adverse conditions as they travel through the space between the peritrophic matrix and the intestinal wall towards the hindgut for subsequent excretion. During this period, the virus would have to face an array of antimicrobial peptides (mostly targeted at bacteria), endo- and exopeptidases, lipases, lysozymes, and amylases with antiviral activity secreted in the anterior midgut [49], as well as serine proteases [56,57] and possibly other antiviral enzymes secreted in the central and posterior sections of the midgut [58,59].

The different sections of the lepidopteran midgut also vary markedly in pH, from close to pH 7 at the entry and exit points to pH 10–12 in the central section [60]. This alkalization arises from the carbonic anhydrase content of goblet cells in the anterior and central sections, whereas alkalization is weakest in the posterior section of the midgut [61]. However, the pH value of the lumen close to the epithelium is 2.5–3 pH units lower than that of the food bolus surrounded by the peritrophic matrix [62]. As such, OBs released from midgut cells may find a refuge from high pH if they are released in the posterior midgut or remain close to the epithelium during their transit along the midgut. OBs released in the posterior midgut will also have a shorter duration of exposure to adverse midgut conditions before reaching the hindgut. Alternatively, ODVs released from OBs under alkaline conditions may remain viable in feces and may have been detected in our bioassay, although their persistence on leaf surfaces would be less than that of ODVs within OBs. To investigate whether viral OBs would withstand these conditions without degrading we attempted to visualize OBs in insect feces by scanning electron microscopy. The resulting images were suggestive of the presence of intact OBs (Supplemental Figure S2), but many other structures were also observed in samples that led us to consider these studies as inconclusive, and we did not pursue this further as efforts to purify OBs largely failed due to fecal contamination.

This little recognized fecally mediated transmission strategy of certain nucleopolyhedroviruses resonates with that of other insect viruses that depend exclusively on the infection of the larval midgut and the production of contaminated feces to achieve transmission, most obviously the cypoviruses (*Reoviridae*) [63], and the viral flacherie diseases caused by Bombyx mori infectious flacherie virus (*Iflaviridae*) and Bombyx mori densovirus (*Parvoviridae*) [64]. It is clear then that the midgut of certain lepidopterans, including *S. frugiperda*, is a source of OB release into the environment for pre-death transmission. Future studies should aim to determine whether this is an ecologically relevant source of virus inoculum at the population level compared to the post-mortem release of OBs. The answer to this question may depend on the stage-dependent feeding habits of the host insect and the relative contribution of inoculum derived from feces compared to that of other environmental sources, such as OBs transported from plant crevices or the soil reservoir.

## Data Availability

The raw data supporting the conclusions of this article will be made available by the authors on request.

## Supplementary Materials

The following supporting information can be downloaded at https://www.mdpi.com/article/10.3390/v17030298/s1, Figure S1: Results of qPCR analysis of feces from infected fourth instars of *Spodoptera frugiperda*; Figure S2: Electron photomicrograph of occlusion body-like structures in feces samples.
