# Supplementary material for: Fecal Transmission of Spodoptera frugiperda Multiple Nucleopolyhedrovirus (SfMNPV; Baculoviridae)
Source: Viruses. 2025 Feb 21;17(3):298. doi: 10.3390/v17030298 (PMC11946685; doi:10.3390/v17030298)
Supplement: Supplementary file 1 [file viruses-17-00298-s001.zip › viruses-3479777-supplementary.pdf]

Supplemental material: Fecal transmission of Spodoptera frugiperda multiple nucleopolyhedrovirus (SfMNPV; *Baculoviridae*)

A.

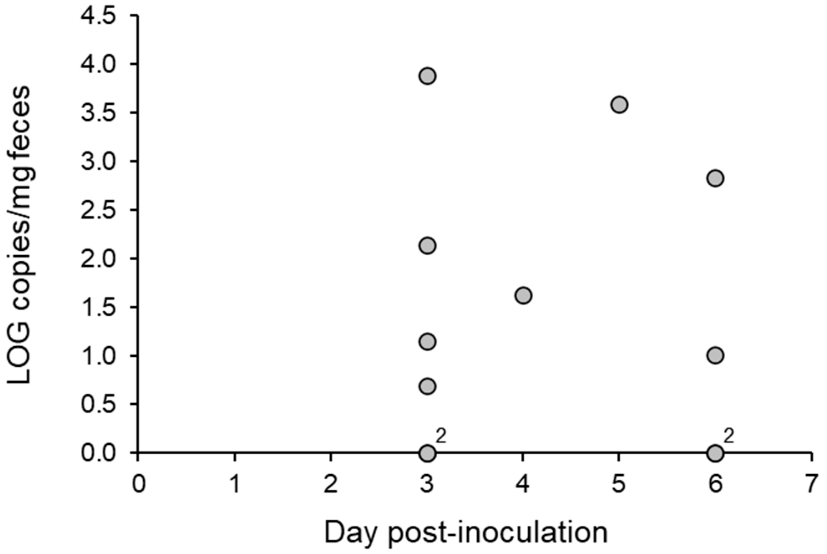

B.

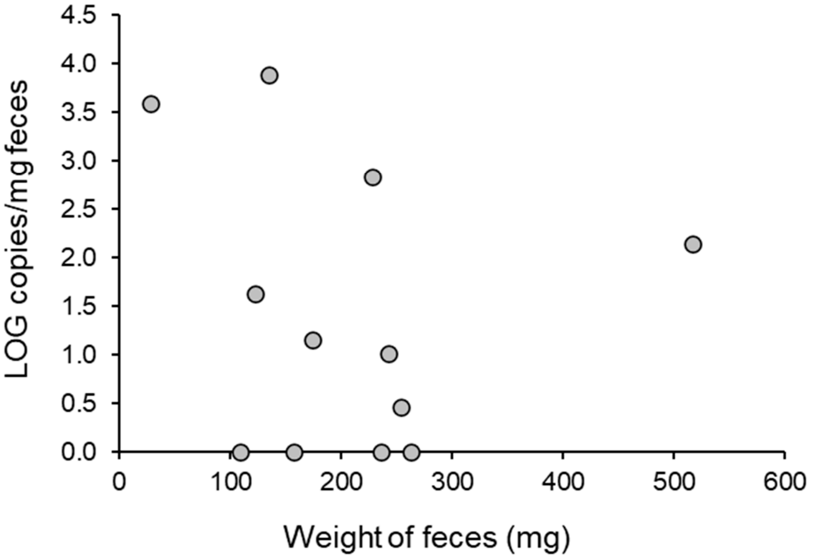

Figure S1. Results of qPCR analysis of feces from infected fourth instars of *Spodoptera frugiperda*. (A) Relationship between log[viral copies/mg feces] and sample time (days post-inoculation). (B) Relationship between log[viral copies/mg feces] and weight of feces in sample (mg). Numbers next to points indicate overlapping data points in A.

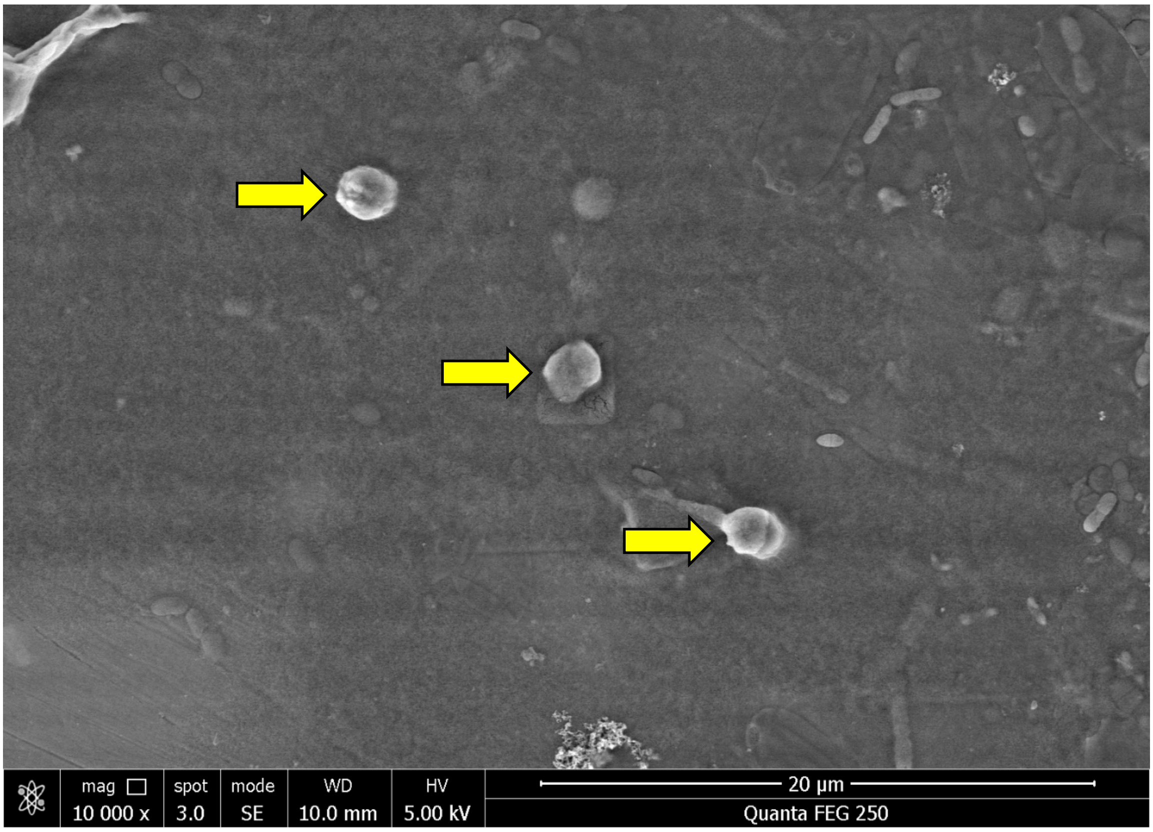

Figure S2. Electron photomicrograph of occlusion body-like structures in feces samples. Structures that were consistent with the appearance of viral occlusion bodies (arrows) were observed in samples of feces collected from *Spodoptera frugiperda* fourth instar larvae at 5 days post-inoculation.
